# Rational Identification of a Colorectal Cancer Targeting Peptide through Phage Display

**DOI:** 10.1038/s41598-019-40562-1

**Published:** 2019-03-08

**Authors:** Débora Ferreira, Ana P. Silva, Franklin L. Nobrega, Ivone M. Martins, Catarina Barbosa-Matos, Sara Granja, Sandra F. Martins, Fátima Baltazar, Ligia R. Rodrigues

**Affiliations:** 1 Centre of Biological Engineering, University of Minho (CEB), Campus de Gualtar, 4710-057 Braga, Portugal; 2 MIT-Portugal Program, Lisbon, Portugal; 3 Present Address: Department of Bionanoscience, Kavli Institute of Nanoscience, Delft University of Technology, Van der Maasweg 9, 2629 HZ Delft, Netherlands; 4 Life and Health Sciences Research Institute (ICVS), School of Health Sciences, University of Minho, Campus de Gualtar, 4710-057 Braga, Portugal; 5 ICVS/3B’s - PT Government Associate Laboratory, Braga/Guimarães, Portugal; 6 Surgery Department, Coloproctology Unit, Braga Hospital, Braga, Portugal

## Abstract

Colorectal cancer is frequently diagnosed at an advanced stage due to the absence of early clinical indicators. Hence, the identification of new targeting molecules is crucial for an early detection and development of targeted therapies. This study aimed to identify and characterize novel peptides specific for the colorectal cancer cell line RKO using a phage-displayed peptide library. After four rounds of selection plus a negative step with normal colorectal cells, CCD-841-CoN, there was an obvious phage enrichment that specifically bound to RKO cells. Cell-based enzyme-linked immunosorbent assay (ELISA) was performed to assess the most specific peptides leading to the selection of the peptide sequence CPKSNNGVC. Through fluorescence microscopy and cytometry, the synthetic peptide RKOpep was shown to specifically bind to RKO cells, as well as to other human colorectal cancer cells including Caco-2, HCT 116 and HCT-15, but not to the normal non-cancer cells. Moreover, it was shown that RKOpep specifically targeted human colorectal cancer cell tissues. A bioinformatics analysis suggested that the RKOpep targets the monocarboxylate transporter 1, which has been implicated in colorectal cancer progression and prognosis, proven through gene knockdown approaches and shown by immunocytochemistry co-localization studies. The peptide herein identified can be a potential candidate for targeted therapies for colorectal cancer.

## Introduction

Colorectal cancer (CRC) is the third most commonly diagnosed cancer worldwide and the second leading cause of cancer-related deaths^1^. The initiation and progression of benign adenoma to malignant adenocarcinoma may be driven by the accumulation of several gene mutations and epigenetic modifications^2^. Early stage screening of CRC can potentially reduce both the incidence and mortality from this type of cancer. However, due to limitations of the current screening modalities in CRC (colonoscopy, biopsy and blood tests), several efforts are being conducted to discover new biomarkers that could be used as alternative screening tools for early diagnosis. Amongst these, peptide ligands that specifically recognize cell surface receptors are particularly promising and are being extensively used in cancer research. Peptides have become an attractive alternative, as they are easy to synthesize in large amounts and their smal size improves tissue penetration, with less nonspecific uptake by the reticuloendothelial system^3^. Moreover, they can be chemically modified to alter affinity, charge, hydrophobicity, stability, and solubility and have been used to functionalize different nanosystems for improved and targeted therapy^4^.

Peptides can be selected in a relatively cost-effective manner using phage display^5–9^. This powerful technology was first introduced in 1985^10^ and has been modified to a rapid high-throughput one step method - Biopanning and Rapid Analysis of Selective Interactive Ligands (BRASIL)^11^, which has enabled the construction of a large number of phage peptide libraries, with a wide range of applications^12–15^. The phage display screening methodology has been exploited and applied in cancer progression and used in selection processes, not only on solid primary tumors, but also on tumor vasculature, metabolism, cell signaling targets and metastasis^7,16,17^. Furthermore, bioinformatics tools and webservers have proven useful to validate and characterize these novel ligands^18^.

Herein we used phage display to identify a peptide, RKOpep, that specifically binds to the cell surface of the human CRC cell lines RKO, Caco-2, HCT 116 and HCT-15, as well as to colorectal cancer tissues. Monocarboxylate transporter 1 (MCT1) was suggested as a possible target based on a bioinformatics analysis and it was further confirmed by gene downregulation approaches and immunocytochemistry co-localization studies. Our results propose a novel targeting system for CRC diagnosis and/or treatment.

## Results

### Specific enrichment of RKO-binding phages

A total of four rounds of selection with RKO cells were performed through *in vitro* biopanning, followed by a negative selection step against normal colon CCD-841-CoN cell line. In each round, the phages that specifically bound to target cells were recovered and used for the next round of selection. In the three initial rounds of selection, the obtained phage pool was not amplified between rounds. However, due to loss of phage concentration, the phage particles obtained in the last two biopanning rounds were amplified using an engineered *E. coli* JM109^+^ strain to minimize the presence of biased sequences^19^. The phage enrichment rate (output/input phage concentration) was gradually increased during the selection rounds, reaching a 45-fold increase at the final round (Table 1).

**Table 1:** Enrichment of RKO cell-bound phages for each round of selection.

Rounds	Input phages (PFU/mL)	Output phages (PFU/mL)	Ratio (output/input)
1	1.0 × 10^11^	2.5 × 10^8^	2.5 × 10^−3^
2	2.5 × 10^8^	6.1 × 10^5^	2.44 × 10^−3^
3	6.1 × 10^5^	1.0 × 10^4^	1.64 × 10^−2^
4	1.1 × 10^10^	6 × 10^8^	5.5 × 10^−2^
5 (Negative Selection)	1.0 × 10^10^	1.1 × 10^9^	1.1 × 10^−1^

### DNA sequencing

After five rounds of biopanning, RKO cell-bound phages were grown to obtain single plaques from which individual phage clones were amplified and genomic DNA extracted. Ten individual clones from rounds 3, 4 and 5 were sequenced and the amino acid sequences deduced and aligned using Clustal Omega (Table 2). Most of these clones displayed a wild type sequence, while the others had distinct peptide hits in which the sequences CKTPNGHLC (RKO-R5-2) and CPKSNNGVC (RKO-R5-1) appeared twice (Table 2). Similar sequences were aligned resulting in the identification of small motif sequences. For instance, lysine (Lys) appeared in the fourth position in 5 of the 13 peptide sequences. Moreover, proline (Pro) appeared eight times at the sixth position, asparagine (Asn) was present six times at the seventh site, and glycine (Gly) appeared in 5 sequences at the eighth position, suggesting a selective pressure for binding to a cell surface epitope.

**Table 2:** Multiple sequence alignment of peptides identified as candidates in rounds 3, 4 and 5 of biopanning generated by Clustal Omega.

Sequence ID	Sequence	Round
RKO-R3-1	C--IAVPSNL-C	3
RKO-R3-2	C---KTPNGHLC	3
RKO-R3-3	C---KXPKGHLC	3
RKO-R4-1	CRPTYSPS---C	4
RKO-R4-2	C-KIHSSET--C	4
RKO-R4-3	C--PKSNNGV-C	4
RKO-R4-4	C--IGNSNTL-C	4
RKO-R5-1	C--PKSNNGV-C	5
RKO-R5-2	C---KTPNGHLC	5
RKO-R5-3	CQSISTAH---C	5
RKO-R5-4	C-NDDVPNK--C	5
RKO-R5-5	C---EIPGKVVC	5
RKO-R5-6	C--LRTPANH-C	5

### Binding assays of phage clones towards RKO cells by cell-based ELISA

**Figure 1:**
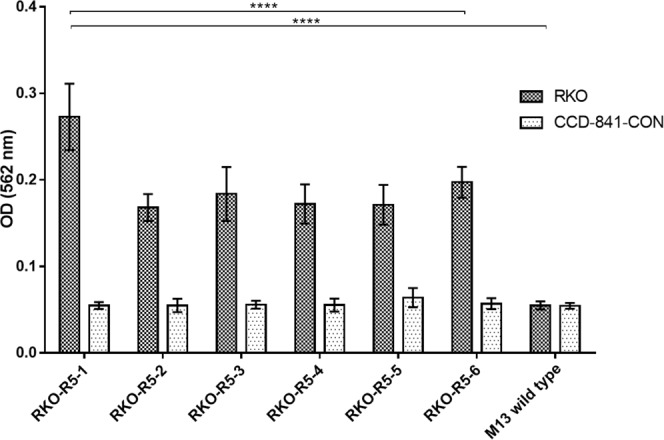
Assessment by cell-ELISA of the binding selectivity to RKO and CCD-841-CoN cells of six phage clones from the last round of biopanning. M13 wild type phage without any displayed peptide was used as negative control. All data are expressed as the mean ± SD of three independent experiments. Two-way ANOVA indicates statistically significant differences within the group assessed by Tukey post-test and denoted as follows: ****p < 0.0001.

A cell-based ELISA assay was used to evaluate the binding capacity to RKO cells of different phages selected after the last round of biopanning (Fig. 1). This experiment excluded false positive colonies, with binding affinity similar to that of the wild-type phage (*p* < 0.0001), and assessed binding selectivity by comparing the binding abilities of each phage clone to RKO and CCD-841-CoN cells. No significant binding of the six phage clones to the control cells CCD-841-CoN was found, suggesting the selectivity of all clones to the RKO cells. Of these, the clone RKO-R5-1 (CPKSNNGVC) showed the highest binding affinity (*p* < 0.0001) being selected for further characterization.

### Peptide analysis

To accurately eliminate the existence of target unrelated peptides (TUPs), false positives and already existing mimotopes, the peptide sequence CPKSNNGVC was analyzed using some tools from Scanner and Reporter of Target-Unrelated Peptides (SAROTUP) that is the most extensive bioinformatics source for assessing peptides derived from phage display selections (Supplementary Table S1). No TUP hit sequences similar to the peptide described in this work were identified being more likely to be a true binder with high specificity to the target. Moreover, the identified peptide was predicted not to bind to contaminants or other components of the screening system (selection-related TUP) and also not to be a clone with growth advantage (propagation-related TUP)

### *In vitro* targeting of RKOpep to human colorectal cancer

**Figure 2:**
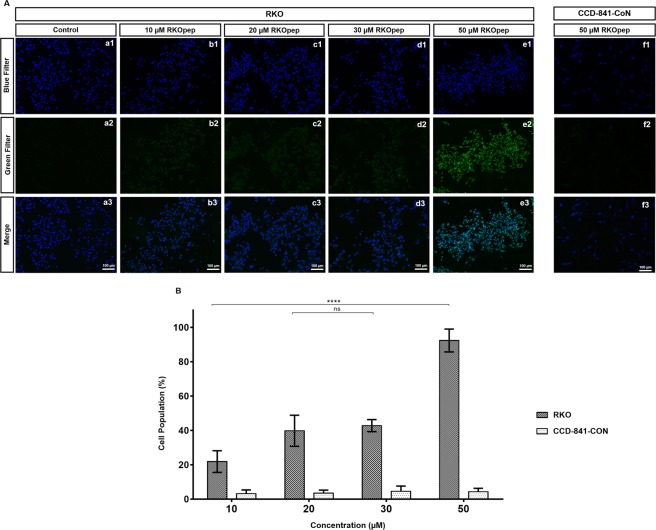
Binding selectivity of FAM-RKOpep towards human colorectal cell line RKO and normal colon cell line CCD-841-CoN demonstrated by fluorescence microscopy and cytometry. (**A**) Microscopy results expressing the binding of increasing concentrations (10 µM–50 µM) of FAM-RKOpep to RKO (b–e) and the highest concentration (50 µM) to CCD-841-CoN (f). The control corresponds to cells only stained with DAPI (a). (1) blue, nuclei stained with DAPI, (2) green, FAM-RKOpep and (3) overlapping of all filters (1 and 2). Scale bars represent 100 μm. (**B**) Flow cytometry results expressing the binding of increasing concentrations (10 µM–50 µM) of FAM-RKOpep to RKO and CCD-841-CoN. All data are expressed as the mean ± SD of three independent experiments. Two-way ANOVA indicates statistically significant differences within the group assessed by Tukey post-test and denoted as follows: ****p < 0.0001 and ns *p* > 0.05.

**Figure 3:**
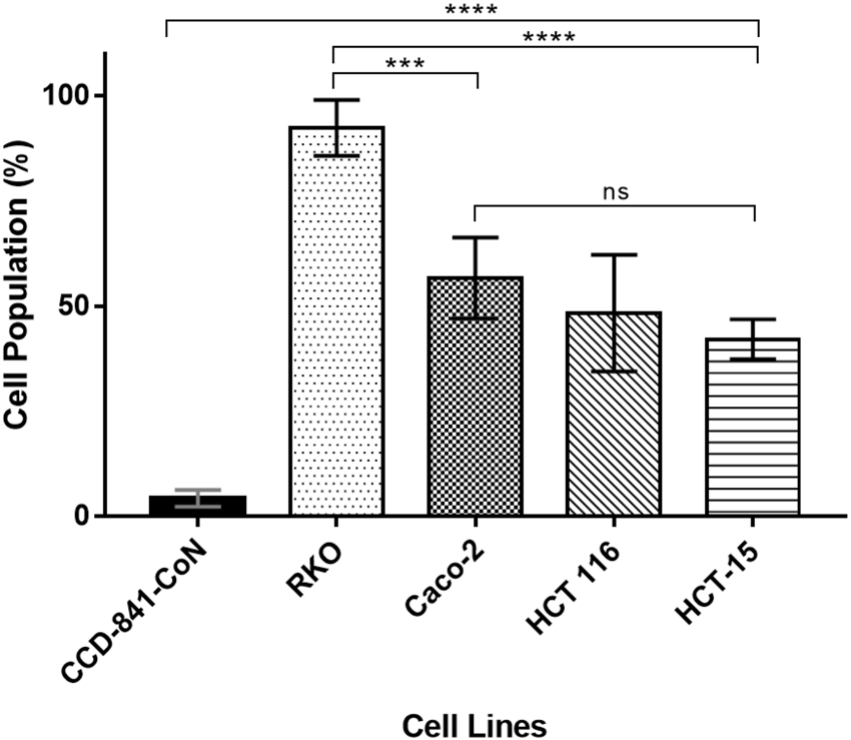
Binding ability of FAM-RKOpep towards human colorectal cell lines demonstrated by cytometry. CCD-841-CoN, RKO, Caco-2, HCT 116 and HCT-15 cells were incubated with the highest concentration (50 µM) of FAM-RKOpep. All data are expressed as the mean ± SD of three independent experiments. Two-way ANOVA indicates statistically significant differences within the group assessed by Tukey post-test and denoted as follows: ****p < 0.0001, ***p < 0.001 and ns *p* > 0.05.

To study whether the free peptide (non-phage-displayed) maintained the binding ability and specificity shown in cell-based ELISA assays, the peptide CPKSNNGVC was synthetized with a FAM label (FAM-RKOpep) at the N-terminus. RKO and CCD-841-CoN cells were incubated with several working concentrations (10 µM, 20 µM, 30 µM and 50 µM) of FAM-labelled peptide and the results were evaluated under fluorescence microscopy and cytometry (Fig. 2A,B). The microscopy and cytometry results are in good agreement, i.e. fluorescence intensity increased with increasing concentrations of FAM-RKOpep in RKO cells in comparison with the control cells. For the higher FAM-RKOpep concentration tested (50 µM), about 90% of the overall RKO cell population was bound by the RKOpep. It is also shown that for CCD-841-CoN, almost no positive signal was detected independently of the peptide concentration, thus confirming the specificity of the peptide RKOpep. In addition, to determine if the FAM-RKOpep specificity was limited to the human CRC cell line RKO, the peptide was incubated with other CRC cells (Fig. 3). A similar binding ability was observed, however, the binding affinity of the peptide at 50 µM was significantly lower for Caco-2 (*p* < 0.001), HCT 116 (*p* < 0.0001) and HCT-15 (*p* < 0.0001) cells when compared to the signal obtained for the RKO cell line. About 56.69 ± 6.8%, 43.29 ± 10.78% and 42.13 ± 4.08% of the overall Caco-2, HCT 116 and HCT-15 cell population was bound to the RKOpep, respectively. These findings proved the successful binding of the peptide RKOpep to different human CRC cells, although with variable binding abilities.

**Figure 4:**
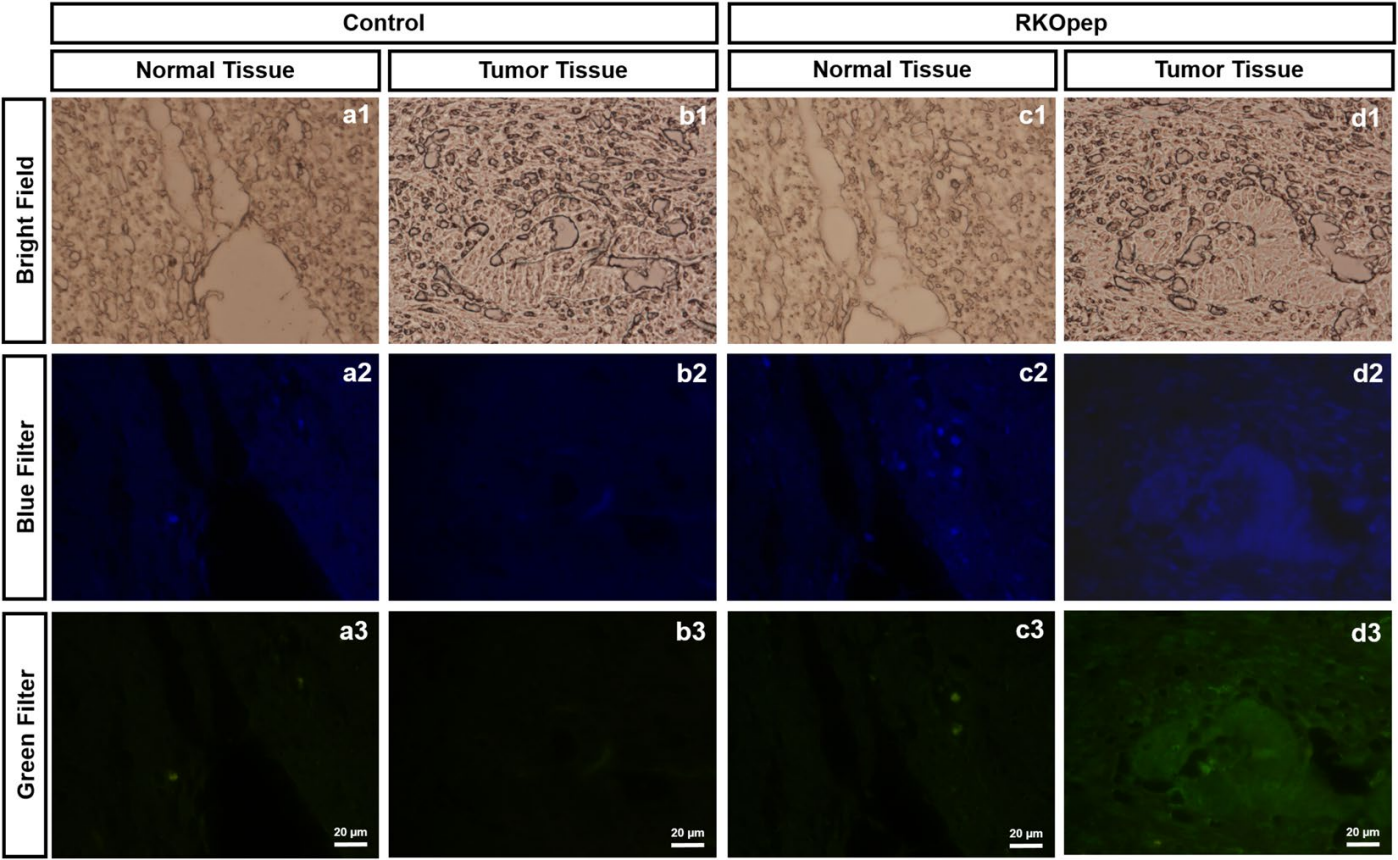
*In vitro* binding affinity of FAM-RKOpep to human colorectal cancer tissues through immunofluorescence staining. Control stands for experiments run without FAM-labelled RKOpep for normal (**a**) and cancer (**b**) tissues; RKOpep stands for experiments run with RKOpep for normal (**c**) and cancer (**d**) tissues. (1) bright field, (2) blue, nuclei stained with DAPI and (3) green, FAM-RKOpep. Scale bars represent 20 μm.

The tumor affinity of FAM-labelled RKOpep was also assessed using human colorectal cancer tissues through immunofluorescence staining (Fig. 4). Visible FAM signal, represented in green, resulting from the incubation of FAM-RKOpep was detected in tumor tissues (n = 3). Using the same microscopy fluorescence settings after acquiring the images, a weak fluorescence for normal-adjacent tissues was observed, thus validating the specificity and the binding capacity of the peptide herein selected by the BRASIL method.

### Bioinformatics identification of the potential target for RKOpep

A structural bioinformatics approach was used to identify the potential targets of RKOpep. A complete overview of all cell surface proteins of five CRC cell lines, including RKO, Caco-2 and HCT 116, was provided by de Wit and collaborators^20^. Using a high-resolution shotgun proteomics analysis, a total of 2609 proteins were identified in the cell surface fractions. By combining additional selection criteria including the presence in three of the cell lines in study, overexpression in carcinomas when compared to adenomas and subcellular location at the plasma membrane, about 59 candidate proteins for the RKOpep were identified.

**Figure 5:**
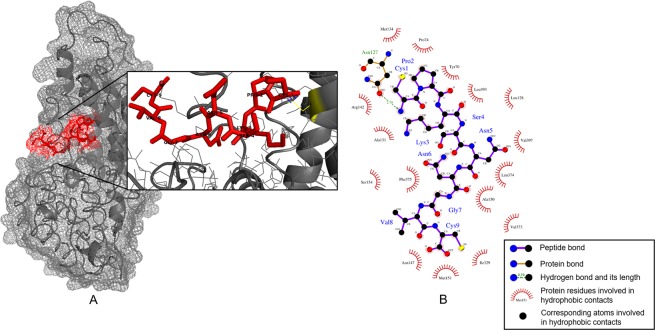
Binding mode of RKOpep with MCT1. (**A**) Most favorable pose of 3D interaction between the RKOpep and the MCT1 (by Pymol). This model was generated by CABS-dock, based on the PHYRE2 predicted structure of MCT1. The RKOpep is shown as a red stick model. MCT1 is represented in grey with key residues shown in yellow. Blue dashed lines represent the hydrogen bonds. (**B**) Two-dimensional interaction between the RKOpep and the MCT1 (by LigPlot). Residues with purple bonds in blue labels are from the RKOpep; residues with brown bonds in red labels are from MCT1. The green dashed line represents a potential hydrogen bond and its length. Hydrophobic interactions are represented by arcs and radiating spokes.

The peptide RKOpep was analyzed using protein-protein Basic Local Alignment Search Tool (BLAST) algorithm for homology to known colorectal cancer-related proteins. According to BLAST results, RKOpep potentially recognizes the membrane protein MCT1 that is one of the 59 candidate biomarkers that the proteomic analysis described by de Wit and coworkers^20^ retrieved after applying selection criteria. The human protein target MCT1 was found with an overall identity of 100%, 0% gaps and with the highest E-value (Supplementary Table S2). In order to explore possible modes of binding of RKOpep to MCT1, computational docking was performed using the flexible and fully blind protein-peptide docking software CABS-dock^21^. This software uses randomly generated peptide conformations, randomly orients these peptides over the protein surface and refines them using Monte Carlo dynamics^22,23^. The three-dimensional structure of MCT1 was predicted with 100% of confidence by using PHYRE2 software^24^. The best three-dimensional docking model, ranked according to trajectory characteristics, is shown in Fig. 5A. The 2D details of the intermolecular interactions between docked RKOpep peptide and MCT1 using LigPlot+ are shown in Fig. 5B.

**Figure 6:**
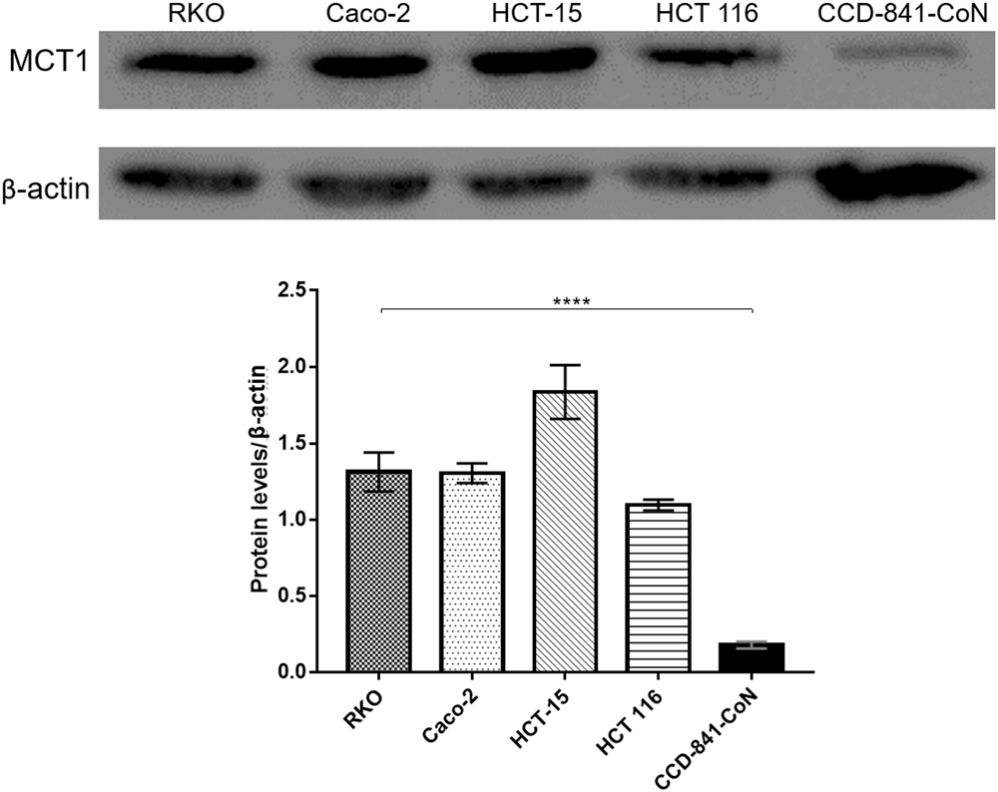
Expression of the MCT1 in colorectal cell lines. Detection of MCT1 (47 kDa) by western blot, in cell lysates of RKO, Caco-2, HCT-15, HCT 116 and CCD-841-CoN. β-actin (42 kDa) was used to confirm equal protein loading. Band intensity was measured using ImageJ software program. Similar results were obtained from three independent experiments and a representative blot is shown.

In order to correlate the binding of RKOpep to the CRC cell lines with the expression of MCT1, its level of expression in the various cell lines under study, including RKO, Caco-2, HCT 116, HCT-15 and CCD-841-CoN, was evaluated through western blot (Fig. 6). Higher levels of MCT1 expression were verified for the CRC cell lines comparing to the normal one (control), being the MCT1 level of expression about 6–10 times superior than the one for CCD-841-CoN cells, according to band intensity calculations using ImageJ.

### Validation of MCT1 as a target of RKOpep

**Figure 7:**
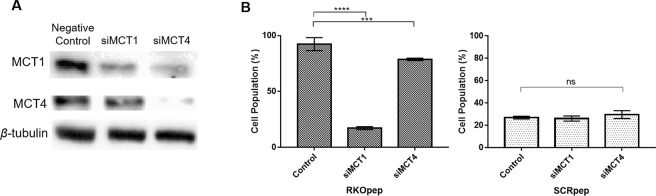
Effect of MCT1 and MCT4 downregulation in RKOpep binding. (**A**) Western blot analysis of MCT1 (47 kDa) and MCT4 (43 kDa) expression in siMCT1 and siMCT4 RKO cells. Cells were transfected with a negative control siRNA, siMCT1, siMCT4 and the expression of MCT isoforms was evaluated after 72 h. β-tubulin (50 kDa) was used to confirm an equal protein loading. Band intensity was measured using Image J software program. Similar results were obtained from three independent experiments and a representative blot is shown. (**B**) Analysis of binding ability of RKOpep and SCRpep to control, siMCT1 and siMCT4 RKO cells, assessed by flow cytometry.

To support and validate the bioinformatics analysis, gene knockdown approaches and immunocytochemistry studies were performed. As observed in Fig. 7, a clear reduction of the fluorescence signal (about 75%) on RKO cells was observed after MCT1 silencing when compared with the control (*p* < 0.0001). To understand the selectivity of RKOpep to MCT1, silencing of another MCT isoform was also performed, namely monocarboxylate transporter 4 (MCT4). A fluorescence signal decrease of approximately 13% was seen after the MCT4 silencing (*p* < 0.001). The scrambled peptide FAM-SCRpep exhibited low binding to the RKO cells and almost the same fluorescence signal, regardless of the conditions tested.

**Figure 8:**
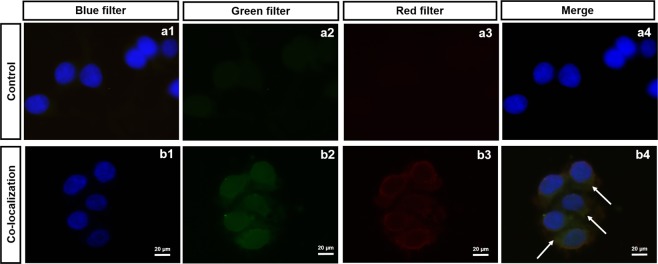
Co-localization of RKOpep with MCT1 by immunocytochemistry. Control represents RKO cells incubated only with DAPI (**a**). Microscopy results expressing the co-localization of the highest concentration (50 µM) of FAM-RKOpep with an anti-MCT1 antibody conjugated with a secondary antibody coupled to Alexa Fluor Plus 680 to RKO cells (**b**). (1) blue, nuclei stained with DAPI, (2) green, FAM-RKOpep, (3) red, Alexa Fluor Plus 680 and (4) overlapping of all filters (1, 2 and 3). Scale bars represent 20 µm.

To demonstrate the RKOpep-MCT1 binding, immunocytochemistry experiments were also conducted. As observed in Fig. 8, the FAM-labelled RKOpep (represented in green) and anti-MCT1 antibody conjugated with a secondary antibody coupled to Alexa Fluor Plus 680 (represented in red) have fluorescence signal on RKO cells. The fluorescence signals overlapped after merging as demonstrated by the yellow/orange color obtained and seemed to co-localize on cell surface, supporting their specific interaction. Control samples were characterized by a faint or no staining, thus strengthening the validation of specific binding of the selected peptide to the MCT1 membrane protein.

## Discussion

CRC is one of the worldwide leading causes of cancer-related morbidity and mortality^25^. Identifying new ligands that specifically target CRC may unravel novel perspectives to develop unique targeted therapies. Human CRC cell lines are useful preclinical model systems as they closely resemble primary tumors^26^. Herein we report the use of a modified phage display methodology to select peptides specific for the CRC cell line RKO. To our knowledge, this study is the first reported in the literature regarding RKO targeting, strongly supporting the need to find new targeting systems for CRC.

RKO-specific peptides were identified by BRASIL using four biopanning rounds of selection against RKO cells and a final negative selection round towards a normal colon cell line, to exclude all peptides that bind non-specifically to cancer cells. To accomplish this, a commercial combinatorial library of seven random amino acids consisting in a disulfide constrained loop was used. This type of disulfide constrained peptide library have been proved useful for the selection of bioactive peptides, disease-specific antigen mimics and/or cell-specific peptides^27–29^.

During phage display, the phage pool between rounds was not amplified since it has been shown that the amplification decreases the library diversity, enriching clones that have an advantage during any of the amplification steps^30,31^. However, due to loss of phage titer, in the last two rounds the obtained phage particles were amplified using an engineered *E. coli* JM109^+^ strain in order to minimize the presence of biased sequences^19^. The phage enrichment rate was progressively increased during the biopanning rounds, clearly indicating that phage clones with binding affinity to RKO cells were enriched (Table 1). This corroborates the expected process of affinity selection, where the specificity of the phage-libraries increases with additional rounds while the diversity drops^30,32^.

Several random clones from rounds 3, 4 and 5 were sequenced being the retrieved peptide sequences analyzed (Table 2). DNA sequencing showed that two peptide sequences appeared two times in sequencing results, CPKSNNGVC and CKTPNGHLC. Afterwards, an *in vitro* cell-based ELISA assay was performed to assess specific binding of the phage clones from the last round of screening to RKO cells (Fig. 1). As a result, RKO-R5-1 phage clone encoding the sequence CPKSNNGVC was the best candidate with the highest binding ability being then selected for further studies.

Although biased sequences were minimized using *E. coli* JM109^+^, the peptide sequence CPKSNNGVC was analyzed to eliminate the possibility of the peptide being a TUP or a false positive (Supplementary Table S1). No relevant similarities were found using SAROTUP between the peptide here identified and those previously published, highlighting the novelty of this peptide.

As RKO-R5-1 phage clone, the free FAM-labelled peptide RKOpep also showed specific selectivity towards RKO cells in comparison to the CCD-841-CoN normal cells. Using microscopy, it was shown for the RKO cells that the fluorescence intensity increased with an increase of FAM-RKOpep concentration, which was not verified for the CCD-841-CoN cells (Fig. 2A). The cytometry analysis also corroborated these results, clearly showing that there is no affinity to the normal cells and that the RKOpep selectively bound to RKO cells (Fig. 2B). Moreover, the FAM-RKOpep showed noteworthy specificity for other three human CRC cells, HCT 116, Caco-2 and HCT-15 (Fig. 3). In addition, the peptide specific targeting of human colorectal cancer tissues, shown by immunofluorescence staining, revealed meaningful results (Fig. 4). Visible and noticeable green fluorescent signal of FAM-RKOpep was detected in human CRC tissues and a weak signal was observed for normal-adjacent ones. This experimental data supports the potential translation of this peptide to clinical oncology. The ability of peptides to recognize human cancer tissues was also demonstrated by Liu and collaborators^33^. They identified novel cancer-specific peptides selected through *in vivo* screening of phage display peptide libraries, that were able to target human cervical cancer tissues.

Identification of the cellular proteins responsible for peptide binding can lead to the discovery of central cellular targets previously unknown, not only by providing the information about the molecules expressed in the pathological state, but also by improving the understanding about what is not expressed under normal physiological conditions^34^. The results described above suggest that RKOpep recognizes a protein that is selectively overexpressed on the surface of at least four human CRC cell lines. Regarding this information, about 59 candidate proteins for the RKOpep were identified using a complete set of cell surface proteins of five CRC cell lines, including RKO, Caco-2 and HCT 116, described by de Wit and collaborators^20^.

To narrow this list of candidate proteins, a multiple sequence alignment for known CRC-related proteins was performed, leading to the identification of two potential targets (Supplementary Table S2). According to this homology search query, RKOpep was found to recognize MCT1, one of the 59 candidate proteins retrieved from the cell surface proteomic analysis after applying the selection criteria.

MCT1 catalyzes the proton-linked movement of many monocarboxylates, such as lactate and pyruvate, across the plasma membrane, and is the major transporter involved in lactate influx into tumor cells^35,36^. In addition to the important functional role of MCT1 in tumor metabolism, increased levels of MCT1 have been implicated in disease progression and prognosis in several human malignancies^37–39^ including CRC^40^. MCT1 has a pivotal role in CRC maintenance and supports its use as a biomarker in primary and metastatic CRC^41^, which makes it a promising target for the novel RKOpep peptide herein identified.

MCT1 has a classical 12 transmembrane-helix structure with intracellular C- and N-terminus and a large cytosolic loop between transmembrane helices 6 and 7^42^. Although no 3D X-ray crystal structure is available for MCT1, its structure was predicted using PHYRE2. The docking results of RKOpep-MCT1 clearly indicated a predominance of hydrophobic interactions over hydrogen bonds (Fig. 5). The amino acid residues of RKOpep defined intensive hydrophobic interactions with the protein residues Met134, Pro74, Tyr70, Leu393, Leu128, Val397, Leu374, Ala150, Val373, Ile329, Met151, Asn147, Phe375, Ser154, Ala131 and Arg142. The majority of these hydrophobic interactions were established with amino acids located at the transmembrane helices of MCT1. In addition, the amino group of RKOpep Cys1 potentially formed a hydrogen bond with the MCT1 Asn127 carbonyl group at 2.70 Å. These preliminary bioinformatics analyses demonstrated that the RKOpep peptide can be specific to MCT1.

The expression level of MCT1 in normal and CRC cell lines was assessed by western blot (Fig. 6). The levels of expression of all CRC cell lines are in good agreement with the binding affinity of the RKOpep to them, as showed by the microscopy and cytometry results. Moreover, the specific recognition of human colorectal cancer tissues by the RKOpep can also be correlated with the overexpression of MCT1 at the plasma membrane of primary CRC tumors, as shown by the immunohistochemistry experiments performed by Martins and coworkers^41^.

To support our bioinformatics analysis, gene knockdown approaches (Fig. 7) and immunocytochemistry (Fig. 8) experiments were conducted using RKO cells. MCT1 silencing led to a significant decrease of the RKOpep binding to RKO cells as compared to the control, thus supporting that the MCT1 transmembrane protein is the target of RKOpep. The MCT family comprises a diverse group of transmembrane proteins. Two members of this family, MCT1 and MCT4, play key roles in the metabolic activity of tissues by mediating the proton-coupled transport of monocarboxylic acids across the plasma membrane^43^. Both MCT1 and MCT4 have been widely studied in colorectal cancer cells^44,45^.

To understand the binding selectivity of RKOpep to MCT1 membrane protein, the knockdown of MCT4 was also evaluated. A low reduction of RKOpep binding ability to RKO cells was observed, probably due to the fact that the siMCT1 had an impact in the MCT4 protein level as observed in the western blot (Fig. 7).

A co-localization of the FAM-labelled RKOpep and the anti-MCT1 antibody was seen, proven by the yellow/orange color obtained, thus confirming the affinity and specificity of the phage display selected peptide to the membrane protein MCT1 and therefore, attesting its usefulness for CRC diagnostics. Geng and collaborators also used immunocytochemistry staining to show that epidermal growth factor receptor 2 (HER2)-specific peptides, obtained through a combination of informatics approaches, including molecular dynamics modeling, co-localize with HER2^46^.

In conclusion, based on the binding of RKOpep to more than one CRC cell line and its ability to specifically recognize human colorectal cancer tissues, we reasoned that this peptide would be a valuable tool for CRC targeting, useful in new diagnostics and/or treatment approaches.

## Materials and Methods

### CRC primary tumor human samples

Tissue samples from 67 patients treated in Hospital de Braga, North of Portugal, between 1^st^ January of 2005 and 1^st^ January of 2010 with CRC diagnosis were collected prospectively. Tumor localization was recorded and classified as colon and rectum (between anal verge and 15 cm at rigid rectoscopy). The histological type of CRC was classified by an experienced pathologist. CRC samples were included into tissue microarrays (TMAs). Prior to TMA construction, haematoxylin and eosin sections were reviewed to select representative areas of the tumor. Normal-adjacent tissue was also included in the TMAs for primary tumors. Each case was represented in the TMA by at least two cores of 0.6 mm. Tissue cores of kidney were used as controls for TMA orientation. The study protocol was approved by the Ethics Committee of Hospital de Braga. The data of CRC series was collected prospectively, patients were informed and signed a written consensus for collecting data and samples collection. All experiments were performed in accordance with relevant guidelines and regulations.

### Cell Lines and Cell Culture

The human epithelial CRC cell line RKO (ATCC CRL 2577), normal colon cell line CCD-841-CoN (ATCC CRL 1790) and CRC cell line Caco-2 (ATCC HTB-37) were cultured in Dulbecco’s Modified Eagle Medium (DMEM, Biochrom). The human CRC cell lines HCT 116 (ATCC CCL-247) and HCT-15 (ATCC CCL-225) were grown in Roswell Park Memorial Institute Medium 1640 (RPMI, Biochrom) supplemented with 10% (v/v) fetal bovine serum (FBS, Biochrom) and 1% (v/v) penicillin-streptomycin (Biochrom). Cells were maintained at 37 °C with 5% CO_2_.

### BRASIL

A commercial phage display library, Ph.D. – C7C, of seven random amino acids flanked by a pair of cysteine residues fused to the minor coat protein pIII of the M13KE phage from New England BioLabs (NEB), was used. An adaptation of the BRASIL method using RKO cells as target^11^ was followed as described elsewhere^27^. The biopanning procedure was repeated four times to enrich the selected phages. A final negative selection step was performed with the normal CCD-841-CoN cell line.

### Selection and amplification of positive clones

Single-stranded DNA (ssDNA) of individual phage clones from rounds 3, 4 and 5 was isolated according to a standard protocol described in^27,47^ and amplified as detailed in^48^.

### DNA sequencing

Before sequencing, the DNA was purified using the Illustra ExoProStar 1-Step kit (GE Healthcare). Sequencing was carried out by GATC Biotech using the M13-pIII primer 5′- TTAACTCCCTGCAAGCCTCA-3′. The SnapGene Version 1.1.3 (GSL Biotech) was used for peptide analysis. The corresponding peptide similarities were identified by Clustal Omega analysis^49^.

### Binding assays using ELISA

RKO and CCD-841-CoN cells were plated in 96-well plates 24 h prior to the addition of positive phage clones at a concentration of 1 × 10^10^ PFU/well and incubated at 4 °C for 1 h. The plate was washed three times with PBS 1X with Tween 20 (PBS-T) prior to incubation with horseradish peroxidase (HRP)-conjugated anti-M13 monoclonal antibody (GE Healthcare), diluted in 1% of bovine serum albumin (BSA) in PBS 1X to the final dilution of 1:5000, and incubated at room temperature for 1 h. Subsequently, the plate was washed with PBS-T three times and the freshly prepared o-phenylenediamine dihydrochloride (OPD, Thermo Scientific) substrate for HRP detection was added to each well and incubated for 15 min. The plate was read at 450 nm on an automated ELISA plate reader (Biotech Synergy HT). The M13KE wild-type phage was used as a negative control. Triplicate measurements were performed at each data point and the signals obtained for each condition were compared.

### RKOpep

The translated peptide sequence (RKOpep) from the phage DNA sequence giving the best binding results in ELISA was synthetized with a N-terminal modification (5/6 FAM), using the latest FMOC solid-phase technology by Thermo Scientific Custom Peptide Synthesis Service. A scrambled peptide (SCRpep) was also synthesized as a negative control. Both peptides were HPLC purified to a purity >99%, lyophilized and analyzed using matrix-assisted laser desorption/ionization mass spectrometry (MALDI-MS) to confirm its molecular mass.

### Binding Assays

For fluorescence experiments, RKO and CCD-841-CoN cells were seeded into plastic coverslips (SPL Life Sciences). After 24 h, cells were fixed with 4% paraformaldehyde (PFA, Sigma) in PBS 1X (w/v) at room temperature for 40 min. After rinsing with PBS 1X, cells were then blocked with PBS 1X containing 1% of BSA for 30 min. FAM-RKOpep was serially diluted in PBS 1X from 0 to 50 µM and incubated with human colorectal cells for 1 h at 4 °C. After being washed with PBS 1X, the coverslips were stained with Vectashield mounting media containing 4′,6-diamidino-2-phenylindole dihydrochloride (DAPI, Vector Laboratories) solution at a concentration of 10 μg/mL, to counterstain the cell nucleic. The images were acquired by an Olympus BX51 microscope incorporated with a high-sensitivity camera Olympus DP71 at 10X magnification. For cytometry assays, a similar protocol was followed. RKO, CCD-841-CoN, Caco-2, HCT 116 and HCT-15 cells were scrapped and recovered for flow cytometry analysis performed with an Epics XL (Beckman Coulter) and at least 20 000 events were counted. Data were analyzed using the Flowing software (version 2.5.1).

### Immunofluorescence staining

TMAs were used to confirm the ability of RKOpep to specifically recognize human CRC tissues. The slides were deparaffinized, rehydrated and after antigenic retrieval, that was performed by heating slides in 10 mM sodium citrate buffer pH 6.0 (Sigma) at 95 °C for 20 min and then slow cooling at room temperature for about 20 min, the tissue slides were blocked with TBS 1X with Tween 20 (TBS-T) for 30 min at 4 °C. Afterwards, the slides were incubated with FAM-RKOpep (50 µM) for 1 h at 4 °C and washed with TBS-T. Finally, the slides were stained with Vectashield mounting media containing DAPI solution. Images were acquired by an Olympus BX51 microscope incorporated with a high-sensitivity camera Olympus DP71 at 40X magnification.

### Bioinformatics analysis

To determine the existence of potential false positives, target unrelated peptides (TUPs), multiple occurrences and already existing mimotopes, several web-based tools were used including BLAST and SAROTUP^50^. Next, to identify the receptor recognized by the peptide, high-resolution cell surface proteomics data was used^20^. The RKOpep peptide sequence was analyzed for homology to proteins with known or putative CRC correlations. The query was performed using the BLAST program (version BLAST+ 2.7.1) against the *Homo sapiens* non-redundant protein database using Blastp (BLASTp with word size of 3 and Blosum62 matrix, http://blast.ncbi.nlm.nih.gov/)^51^. Furthermore, molecular docking was performed to study peptide-protein interactions. The MCT1 structure used for peptide-docking analysis was predicted by homology modeling using PHYRE2 software^24^. Computational docking analysis, without a previous knowledge about the binding site, was performed using CABS-dock web server with the default parameters^21^. LigPlot + program (version 1.4.5) was used to illustrate schematic two-dimensional representation of peptide–protein interaction^52^. PyMol (version 2.0.7) was employed for the display and analysis of molecular structures.

### Western blotting

For western blot, about 20 µg of total protein were loaded, separated by 10% sodium dodecyl sulfate-polyacrylamide gel electrophoresis and transferred onto a nitrocellulose membrane (Protan Amersham, GE Healthcare). Then, to avoid non-specific interactions, the membrane was blocked with 5% BSA in TBS-T for 30 min at room temperature and then exposed overnight at 4 °C to the primary antibodies (dilution in TBS-T with 5% BSA) MCT1 (1:200 dilution, H-1, sc-365501, Santa Cruz Biotechnology), MCT4 (1:500 dilution, G-7, sc-376465, Santa Cruz Biotechnology), β-actin (1:2000 dilution, #4967S, Cell Signaling Technology) and *β-*tubulin (1:3000 dilution, 605101, Biolegend). After washing in TBS-T, the membranes were incubated with anti-mouse (sc-2031, Santa Cruz Biotechnology) or anti-rabbit (#7074S, Cell Signaling Technology) secondary antibodies linked to HRP (1:5000 dilution in TBS-T with 5% BSA) for 90 min at room temperature, respectively. The bands were detected using a chemiluminescent substrate (Clarity Western ECL Substrate, Bio-Rad) in ChemiDoc XRS + System (Bio-Rad). Band intensity was quantified by ImageJ.

### Downregulation of MCT1 and MCT4 expression

Silencing of MCT1 and MCT4 expression was performed using 5 nM of Silencer Select Pre-Designed & Validated siRNAs from Thermo Fisher (MCT1 siRNA: s580 and MCT4 siRNA: s17417), using an adequate control (Silencer Select Negative Control siRNA: 4390843). Cells were transfected using Lipofectamine RNAiMAX Reagent (Thermo Fisher) according manufacturer’s instructions.

### Immunocytochemistry for co-localization of FAM-RKOpep and MCT1

For immunocytochemistry, approximately 2 × 10^5^ of RKO cells were seeded into plastic coverslips and cultured overnight. FAM-RKOpep was diluted in PBS 1X (50 µM) and incubated with adherent RKO cells for 1 h at 4 °C. After rinsing with PBS, the cells were fixed with 4% of PFA for 40 min and then permeabilized with 0.01% Triton-X (Merck) for 5 min. Afterwards, non-specific binding sites were blocked with TBS-T 1X containing 5% BSA for 30 min at 4 °C, and next exposed overnight at 4 °C to the primary antibody (dilution in TBS-T with 5% BSA) MCT1 (1:200 dilution, AB3538P, Merck). After rinsing with PBS 1X, the cells were incubated with anti-rabbit (#A-11011, Thermo Scientific) secondary antibody coupled to Alexa Fluor Plus 680 (1:500 dilution in TBS-T with 5% BSA) for 90 min at room temperature. After being washed with PBS 1X, the cells were stained with Vectashield mounting media containing DAPI. The images were acquired by an Olympus BX51 microscope incorporated with a high-sensitivity camera Olympus DP72 at 100X magnification.

### Statistical analysis

Data was expressed as the mean ± standard deviation (SD) of three independent experiments. Two-way ANOVA with Tukey post-test was performed using GraphPad Prism (Graph-Pad Prism version 6.00 for Windows), to identify differences among multiple groups, considering a significance level of 95%.

## Supplementary information

Suplemmentary material
