# Supplementary material for: Rational Identification of a Colorectal Cancer Targeting Peptide through Phage Display
Source: Sci Rep. 2019 Mar 8;9:3958. doi: 10.1038/s41598-019-40562-1 (PMC6408488; doi:10.1038/s41598-019-40562-1)
Supplement: Supplementary file 1 — Suplemmentary material [file 41598_2019_40562_MOESM1_ESM.pdf]

Supplementary Information

Rational Identification of a Colorectal Cancer Targeting Peptide through Phage Display

Débora Ferreira, Ana P. Silva, Franklin L. Nobrega, Ivone M. Martins, Catarina Barbosa-Matos, Sara Granja, Sandra F. Martins, Fátima Baltazar and Ligia R. Rodrigues

Table S1 - Validation of the selected peptides through the analysis of existing TUPs, false positives and/or mimotopes.

Tool	Result
MimoScan – **Designed to check if there is any peptide that matches the patterns submitted**	No hits found
MimoSearch – **Designed to check whether peptides have also been gained by other groups with various targets**	No hits found
TUPScan – **Designed to check if the peptide bears any known TUP motif**	No hits found

Table S2 - Search results using NCBI protein–protein blast BLASTP 2.7.1 for the selected peptide sequence. Peptides were analyzed against Homo sapiens non-redundant protein database using BLASTP for cancer-related proteins (PSI-BLAST, word size of 3, Blosum62 matrix and E<10), to identify proteins with homologous motifs.

Peptide Sequence	Homologous protein	Query cover (%)	% Identities (% Gaps)	E-value	Accession Number
**RKOpep CPKSNNGVC**	Monocarboxylate transporter 1	44	100 (0)	1.9	NP_003042.3
	Complement component 5	66	67 (0)	2.7	AAI13739.1

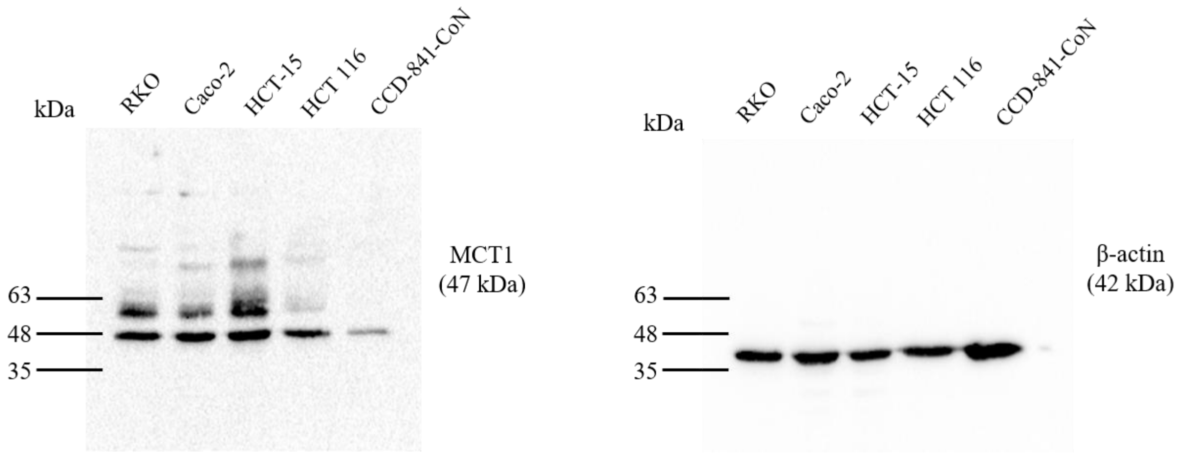

Figure S1 - MCT1 and β-actin staining of the membrane for colorectal cell lines for the western blot data in Figure 6. The nitrocellulose membranes were cut according to protein marker after transfer.

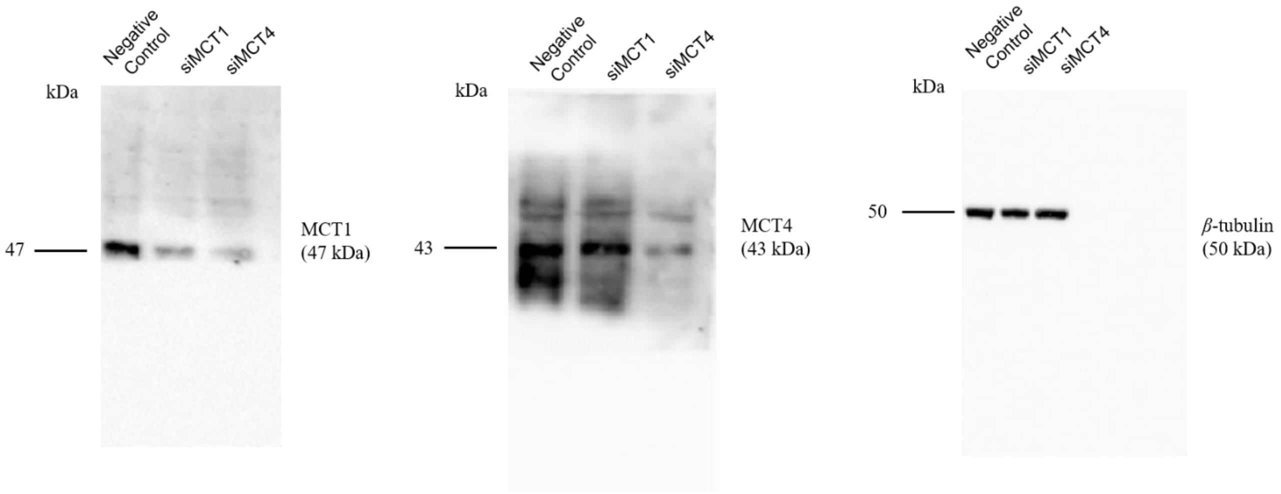

Figure S2 - MCT1, MCT4 and β-tubulin staining of the membrane for negative control, siMCT1 and siMCT4 RKO cell line for the western blot data in Figure 7. The nitrocellulose membranes were cut according to protein marker after transfer.
